# Emission Characteristics of Volatile Organic Compounds from Material Extrusion Printers Using Acrylonitrile–Butadiene–Styrene and Polylactic Acid Filaments in Printing Environments and Their Toxicological Concerns

**DOI:** 10.3390/toxics13040276

**Published:** 2025-04-04

**Authors:** Yuan Gao, Yawei Xue, Chenyang Sun, Luhang She, Ying Peng

**Affiliations:** 1 Instrumentation and Service Center for Science and Technology, Beijing Normal University, Zhuhai 519087, China; yuan.gao@bnu.edu.cn (Y.G.); 202321180070@mail.bnu.edu.cn (Y.X.); 2 Research and Development Center for Watershed Environmental Eco-Engineering, Advanced Institute of Natural Sciences, Beijing Normal University, Zhuhai 519087, China; 15893863095@163.com (C.S.); 15764410923@163.com (L.S.); 3 State Key Laboratory of Wetland Conservation and Restoration, School of Environment, Beijing Normal University, Beijing 100875, China; 4 Key Laboratory of Coastal Water Environmental Management and Water Ecological Restoration, Guangdong Higher Education Institutes, Beijing Normal University, Zhuhai 519087, China; 5 Zhuhai Key Laboratory of Coastal Environmental Processes and Ecological Restoration, Beijing Normal University, Zhuhai 519087, China

**Keywords:** 3D printing environment, material extrusion, FDM, acrylonitrile–butadiene–styrene, polylactic acid, emission characteristics, toxicological concern

## Abstract

The utilization of 3D printing releases a multitude of harmful gas pollutants, posing potential health risks to operators. Materials extrusion (ME; also known as fused deposition modeling (FDM)), a widely adopted 3D printing technology, predominantly employs acrylonitrile–butadiene–styrene (ABS) and polylactic acid (PLA) as printing materials, with the respective market shares of these materials reaching approximately 75%. The extensive usage of ABS and PLA during the ME process leads to significant volatile organic compound (VOC) emissions, thereby deteriorating the quality of indoor air. Nevertheless, information regarding the emission characteristics of VOCs and their influencing factors, as well as the toxicological impacts of the printing processes, remains largely unknown. Herein, we thoroughly reviewed the emission characteristics of VOCs released during ME printing processes using ABS and PLA in various printing environments, such as chambers, laboratories, and workplaces, as well as their potential influencing factors under different environmental conditions. A total of 62 VOC substances were identified in chamber studies using ABS and PLA filaments; for example, styrene had an emission rate of 0.29–113.10 μg/min, and isopropyl alcohol had an emission rate of 3.55–56.53 μg/min. Emission rates vary depending on the composition of the filament’s raw materials, additives (such as dyes and stabilizers), printing conditions (temperature), the printer’s condition (whether it has closure), and other factors. Additionally, we reviewed the toxicological concerns associated with hazardous VOC species commonly detected during the ME printing process and estimated cancer and non-cancer risks for users after long-term inhalation exposure. Potential health hazards associated with inhalation exposure to benzene, formaldehyde, acetaldehyde, styrene, and other substances were identified, which were calculated based on concentrations measured in real indoor environments. This study provides valuable insights for future research on the development of ME printing technologies and offers suggestions to reduce VOC emissions to protect users.

## 1. Introduction

Globally, 3D printing development has been growing rapidly in recent years, and its global market value increased to USD 14 billion in 2020 from USD 7.4 billion in 2017 [1]. This figure is predicted to reach USD 56 billion in 2027 [2,3]. These trends point toward the increasing importance of 3D printing technology in our daily lives, which has been widely used in many industries—for example, manufacturing, building, aerospace and medical devices, and environmental research, as well as common applications in offices, small workplaces, and homes [4,5,6,7].

Material extrusion (ME) printing is a commonly used 3D printing technology due to its ease of operation and the diversity of its printing materials [8]. More importantly, ME printers reduce the cost of creating basic proof-of-concept models and simple prototyping substances in small-scale indoor environments such as homes, studios, and small offices. For example, some small companies have used this technology and saved as much as 50% on their tooling costs. The consumption of filaments for ME printing is increasing annually, with a compound annual growth rate of approximately 23.3% of the total market figures [9,10]. Previous reports have demonstrated that various harmful indoor pollutants, e.g., volatile organic compounds (VOCs) such as styrene, acetaldehyde, and acetone, are primarily emitted from the printing of raw materials (for example, polymer and thermoplastic material) and secondarily from the process of 3D printing (for example, heating) [11]. Additionally, the increase in the use of printing composite materials is related to the increase in the number of volatiles. Some in vitro cell experiments and human and animal exposure experiments have shown evidence of pollutants induced by 3D printing and adverse health impacts [12]. Furthermore, individuals who work with 3D printers for more than 40 h per week are more likely to report respiratory-related asthma or allergic rhinitis [13].

Direct and indirect toxic effects in individuals under occupational exposure to synthetic polymers of ME printing have been observed in previous studies, such as asthma, chronic obstructive pulmonary disease, allergic rhinitis, and DNA damage [13,14,15,16]. ME printing filaments are made from plastic, and some contain metal and ceramic materials [17,18,19]. Plastic was the largest segment in the production of 3D printing filaments, which includes acrylonitrile–butadiene–styrene (ABS), polylactic acid (PLA), nylon, polyvinyl alcohol (PVA), polycarbonate (PC), and high-density polyethylene (HDPE). Due to the outbreak of COVID-19, there is a high demand for medical devices and medical applications, i.e., swabs, face masks, and ventilator splitters, all of which can be produced using ME printing with plastic filaments [19]. As mentioned in the 2021 3D printing industry report, over 50% of the 3D printing materials consumed in China are plastic materials. Among plastics, ABS and PLA filaments are the two most widely used types, accounting for 75% of the market. However, these materials are often not fully pure. For instance, talc is commonly added to improve printability, and additives in these materials may further increase particles and VOC emissions. With the widespread application of ME printing technology in offices, households, and for domestic purposes, it is of great importance to understand the characteristics of ME printers that use ABS and PLA filaments, as well as their associated toxicological concerns. Concerns about particulate and VOC emissions during the ME 3D-printing process and their health impacts have attracted much attention over the years [12,20,21,22,23,24,25,26,27,28,29,30,31,32,33,34,35]. However, there is currently a lack of systematic reviews regarding the consumption of ABS and PLA in ME printer emissions, which hinder our understanding of VOC emissions and secondary formation pollutants, as well as evaluating the potential risks to the environment.

Therefore, this review summarizes the recent progress in studies on the emission characteristics of VOCs released from ME printers using ABS and PLA filaments in various settings, including chambers and workplaces (such as household, educational, and small business environments), over the past decade. Meanwhile, the potential influencing factors of VOC emissions are discussed, and the potential indoor health impacts of hazardous VOC species released from ABS and PLA filaments are also reviewed. In total, over 150 studies were found by searching the following keywords in different online platforms (i.e., Google Scholar and Web of Science): FDM 3D printing, ABS, PLA, emission of volatile organic compounds, hazards, occupational exposure, and risk assessment. The inclusion criteria for choosing publications were as follows: (1) contain a discussion on the VOC emission values from ABS and PLA printing process; (2) contain a discussion about the toxicology of long-term exposure; (3) not workshop and conference papers; and (4) published in the English or Chinese language. We excluded publications that were duplicates and those that did not meet the above criteria. The aim of this study was to understand the potential emission characteristics of harmful chemicals, the influencing factors (e.g., environment parameters and the setup of printers) of emissions of harmful chemicals, and toxicology effects from exposure to harmful chemicals through a comparison of the emitted VOC pollutants during the ME printing process with ABS and PLA filaments. This study provides suggestions and recommendations for the safe development and application of ME printing technology.

## 2. VOC Measurement and Emission Characteristics of ABS and PLA Filaments

According to the literature, VOCs have been collected and analyzed in different ways. Typically, total volatile organic compounds (TVOCs) are measured using a photoionization detector (PID) as concentrations in parts per billion (ppb). Mendez et al. (2017) used the real-time PID method to measure TVOC value in a 0.18 m^3^ stainless steel chamber and concluded that VOCs were traceable pollutants [36]. The individual VOCs most often used Tenax^®^ sorbent tubes or canisters and analyzed with gas chromatography–mass spectrometry (GC-MS). Some studies used DNPH cartridges and analyzed them using a high-performance liquid chromatography-UV detector (HPLC-UV) to identify oxygenated VOCs (OVOCs). Recently, the Proton Transfer Reaction–Mass Spectrometry (PTR-MS) technique has been used for on-line VOC measurement [30]. Potter et al. (2019) [37] discussed VOC emissions under different temperature situations in mass per printed filament (μg/g). The VOC emission concentration was measured in real indoor environments, expressed in units with the unit of mass emitted per volume (μg/m^3^). For example, the TVOC values were 216.5–317.7 μg/m^3^ for ABS filaments in a printing room and university laboratory [38,39]. The dominant VOCs species reported in the real indoor environment were sebacic acid, 3-methylbut-2-enyl propyl ester, decane, styrene, 2-amino-2-oxo-acetic acid, N-[3,4-dimethyl], ethyl ester, and nonanal, and the concentration ranged from 3.0 to 23.0 μg/m^3^ [38,39,40]. These dominant species were mostly OVOCs, which may indicate that the emitted VOCs were being oxidized in the real indoor environment, where the concentration of oxidants (i.e., O_3_ and OH) were likely significant under the light sources.

The VOC emission rate is an important parameter for determining the emission characteristics of different filaments. In most of the chamber studies, the emission rate of VOCs was discussed in terms of the mass emitted per unit time (μg/min). Tables S1 and S2 summarize the emission rates of different VOC substances from ABS and PLA filaments [21,35,36,39,41,42,43,44,45,46,47,48,49,50]. A total of 36 types of VOC, including hydrocarbons, ketones, aldehydes, alcohols, aliphatic hydrocarbons, carboxylic acids, esters, siloxanes, and other compounds, were identified from the emissions when ABS filaments were used as the printing material. The range of these substances varied from 0.01 μg/min to around 113.0 μg/min (Table S1). The predominant VOC species identified was styrene, with emission rates ranging from 0.3 μg/min to 113.0 μg/min, depending on the filament brands, printers, and experiment conditions employed. For example, the emission rates of styrene were reported as 33.5 μg/min and 113.0 μg/min when using white ABS filaments in two different printer brands, FlashForge (China)and MakerBot (U.S.A), respectively [44]. Stefaniak et al. (2017) [41] compared the emission of four color ABS filaments in the same ME printer, and the highest emission was observed in the nature color filament (9.0 μg/min). The comparative emission value was determined for red ABS filament to be 8.5 μg/min, and its emission rate was also shown to be a relatively high value in other studies [21,37,50,51]. Ethylbenzene emerged as the second most abundant VOC species, with emission rates varying between 0.2 μg/min and 5.4 μg/min. The relatively high values were found when using white (5.4 μg/min) and red (4.8 μg/min) color filament [39,41,42,43,44,45,46,47]. The VOC substances mentioned above exhibit toxicity and are associated with significant adverse health effects [12]. Styrene, which has a strong smell, may affect the central nervous system and has been assessed as being possibly carcinogenic to humans (Group 2A) by the International Agency for Research on Cancer (IARC). Ethylbenzene is listed as a possible carcinogen by the IARC, causing adverse effects in the kidneys and testicular tissue [52].

According to Table S2, the emission of VOC substances from PLA filaments is quite different, with the emission value relatively low compared to the emission from ABS filaments. Methyl methacrylate, isopropyl alcohol, lactide, and acrylic acid dimers were reported as primarily emitted from PLA filaments [12]. A total of 37 VOC substances were reported, with emission rates ranging from 0.1 to 56.5 μg/min. Isopropyl alcohol, acrylic acid dimmer (1,4-dioxane-2,5-dione, 3,6-dimethyl-), ethanol, and hexanal were identified as the main VOC species in previous chamber studies [39,44,48]. The highest emission rate (median value of 14.8 μg/min) was detected for isopropyl alcohol, which has been reported as a byproduct of polylactic acid depolymerization. PLA is a biodegradable filament made from renewable organic materials, including corn starch or sugarcane. According to Floyd et al. (2017) [47], PLA-based filaments primarily emit d-limonene and acrylic acid, where acrylic acid dimer may form lactic acid via dehydration and subsequent dimerization. Furthermore, the human body can naturally produce or eliminate lactic acid, and PLA has a relatively low operating temperature, resulting in reduced VOC emissions [53]. D-limonene may potentially prevent some cancers. Consequently, PLA-based filaments are likely safer than ABS-based filaments. However, according to the literature, the emission of VOC substances is not limited to acrylic acid and d-limonene; many more VOC species were identified, which is summarized in Table S2 [21,39,40,41,44,46,48,49,50]. It is also worth noting that the emission of ethanol and octanal from PLA filament reported in many studies is mainly related to the process of print-bed production preparation [54].

**Figure 1:**
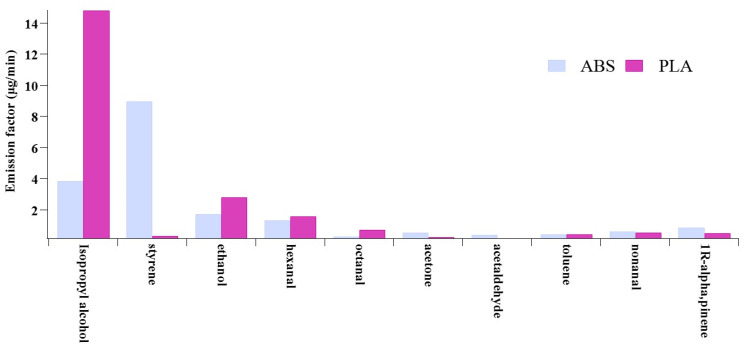
VOC species emissions from PLA and ABS filaments (*n* ≥ 3).

In summary, styrene, a typical primary pollutant, is the most common pollutant emitted from the use of ABS filaments. However, the reported emission rates of styrene exhibit substantial variability across the studies. The emission rates of pollutants from PLA filaments are relatively low, and the most common pollutants emitted are secondary pollutants. In addition, out of the 63 VOCs identified in each filament, only 10 VOC species were consistently detected in both filaments (Figure 1), including isopropyl alcohol, ethanol, and hexanal, among others. Emissions of ethanol, acetaldehyde, and acetone were reported in most studies, suggesting that they may serve as additives and product intermediates of the filaments [21]. The VOC species mentioned above may be secondary pollutants formed during the printing process, but the formation mechanisms are not well understood, so further studies are needed.

## 3. Potential Influences of VOC Emission from ABS and PLA Filaments

In general, the emission of VOCs is closely related to the chemical composition of the filaments. For example, ABS is synthesized by polymerizing styrene and acrylonitrile in the presence of polybutadiene, which can undergo decomposition into styrene, butadiene, and acrylonitrile under high-temperature conditions [55,56,57]. Therefore, styrene, acrylonitrile, and polybutadiene have been reported to be the primary pollutants emitted from ABS filaments [20]. Styrene has consistently been reported to be the predominant VOC emitted from ABS filaments across numerous studies [21,37,41,43,50,51,58,59,60,61]. Furthermore, apart from styrene, ethylbenzene, acetone, and acetaldehyde have been identified as the predominant VOCs emitted. This is attributed to the formation of these three compounds through the thermal degradation of filament monomers [21].

To fulfill various requirements of 3D-printed products, additives such as dyes, plasticizers, and stabilizers are commonly incorporated into filaments [21,22,51,62]. However, the incorporation of these additives in ABS filaments has an impact on the emissions of gaseous pollutants. For example, Potter et al. (2019) [37] compared VOC emissions from ABS filaments and ABS filaments with carbon nanotubes (which are additives that can be used in 3D printers to produce conductive electrical components), and the results showed a decrease in styrene emissions and an increase in the formation of α-methyl styrene in the presence of carbon nanotubes [37]. In addition, benzaldehyde, a hazardous pollutant, exhibited an upward trend in its values due to surface interactions [21,63], as adsorbed oxygen on the surface can increase the oxidation capacity. Furthermore, the addition of ABS filaments may also contribute to the formation of toxic particles. For instance, Zhang et al. (2017, 2018) [64,65] found that the mass spectra of ABS-emitted particles exhibited various compositions that differed from those of the raw filament material monomers, apparently due to the presence of a minor unknown filament additive, leading to particle formation [25,27,64,65,66,67,68].

Regarding PLA, acrylic acid is expected to be the predominant emission [47], but it has not been detected in many studies because acrylic acid readily forms other compounds. The dominant VOC species reported were identified as the major ones listed in Table S2. While various studies have identified complex VOC species [26,39,49,59,60,61,69,70,71,72,73], there is limited information available regarding their emission or secondary formation process. Inkinen et al. (2011) [74] reviewed the possible origins and effects of lactic acid monomers and lactide on the formation of PLA products. They reported that the precursor of PLA, lactic acid fermentation broth, contains many different impurities, such as glycerid, succinic, formic, fumaric, puryvic, methanol, ethanol, butanol, methyl, ethyl, and butyl lactates. These impurities in chemical compounds may be present in PLA products, contributing to VOC emissions during or after the 3D printing process. Meanwhile, the additives of PLA filaments also impact the emissions of gaseous pollutants. Floyd et al. (2017) [47] discovered a higher emission rate of TVOCs when using bronze-PLA filaments compared to pure PLA filaments, which was attributed to the presence of additives. The varied filament brands likely contain different impurities and additives, resulting in variations in VOC emissions and differences in trace components within the bulk material. Therefore, defining the purity of filament is a crucial step to ensure the biodegradability and safety of PLA filaments. Hence, further research is needed to understand the chemical reactions occurring during or after the printing process when using PLA filaments.

Table 1 summarizes the individual VOC emissions from the same-colored ABS filament using different printers in an environmentally controlled chamber. Here, we chose red filaments as an example because more data were available. A total of 21 VOC species were reported, with concentrations ranging from 0.4 to 912.8 μg/m^3^. The identified VOC species varied with different printers. Only two VOCs were detected in three printers: styrene and ethylbenzene; acetophenone was measured in two printers. One interesting finding observed in the three studies (carried out in 2016, 2017, and 2019) was a significant reduction in the emission concentrations of styrene, ethylbenzene, and acetophenone [41,43,44]. In addition, the number of emitted VOC species also decreased. Davis et al. (2019) [21] compared emission rates of TVOC by using five commercial ME printers from various manufacturers, revealing a range of emission rates between 9.0 and 18.2 µg/min. Davis, Stefaniak, and others used the same method to measure the emission rate of TVOCs in 1 m^3^ and 0.5 m^3^ stainless steel chambers using different ME printers, with the emission rates ranging from 13.9 µg/min to 59.2 µg/min [21,71]. These results suggest that the printing brand is the other main factor affecting VOC emissions. However, it is still unclear whether the evolution of 3D printers and filaments will result in a reduction in VOC emissions. The VOC analysis methods used in the studies were also inconsistent, which might have introduced bias into the measurement data. Therefore, future studies should be carried out to identify the effects of printing materials and printers by utilizing standardized experimental procedures.

**Figure 2:**
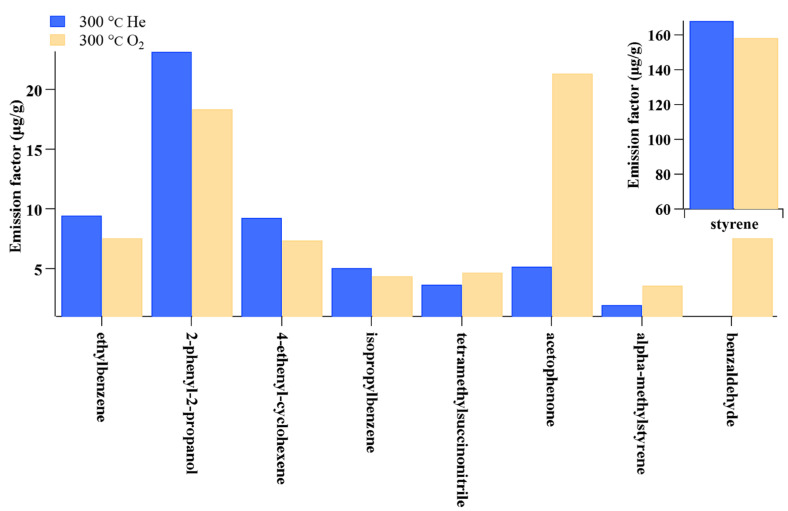
Emission factor of VOC species under He and O_2_ conditions (source: [37]).

The emissions of volatile organic compounds (VOCs) from ME printers that utilize ABS and PLA may also be influenced by environmental conditions, particularly in facilitating the secondary generation of VOCs. For example, benzaldehyde and acetaldehyde are typical oxygenated volatile organic compound (OVOC) species that can be emitted both primarily and secondarily. In the literature, benzaldehyde and acetaldehyde have been detected in ME printing processes using ABS and PLA filaments, which may imply the existence of VOC secondary formation during the printing process. A study by Potter et al. (2019) [37] showed that 3D printing under O_2_ conditions emitted more VOC species than the printing process under He conditions (Figure 2), which supports the existence of secondary formation during the 3D printing process. However, the secondary formation or degradation of carbonyl compounds during the printing process is not fully understood, as only 4-oxopentanal, a carbonyl compound, was reported by [41].

**Figure 3:**
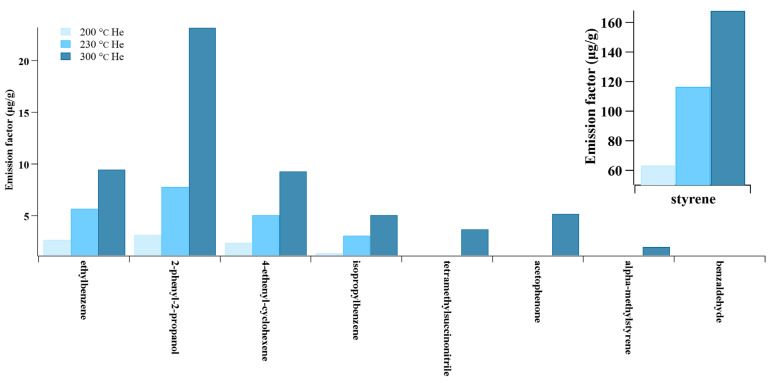
Emission factor of VOCs at different temperatures (source: [37]).

Furthermore, previous studies have demonstrated that the operational temperature is a key influencing factor in the emissions of VOCs from ABS ME printing, with higher temperatures promoting greater VOC production. For example, the TVOC emission rates were found to be 557, 567, and 716 μg/min at a 220 °C, 240 °C, and 260 °C nozzle temperature, respectively [75]. The dominant species, styrene and ethylbenzene, exhibited approximately threefold increases when the temperature was raised from 200 °C to 300 °C [37]. Moreover, elevated temperatures of up to 300°C resulted in the detection of additional VOC species, such as acetophenone and alpha-methyl styrene (Figure 3).

Sunlight is another environmental condition that affects the emissions of VOC species. For example, many studies have reported the emission rates or concentrations of styrene, but only a few studies have identified acrylonitrile and polybutadiene emissions from 3D printing (Tables S1 and S2). Based on the literature, the lack of data on acrylonitrile and polybutadiene emission rates or concentrations is attributed to the emission of secondary products formed during the printing process (resulting from the formation or degradation of primary pollutants). Acrylonitrile is a compound that readily volatilizes in air and can be easily degraded by photooxidation processes [57], while the photooxidation products derived by polybutadiene can result in the formation of hydroxyl, carboxyl, ketone, and epoxy groups of VOCs [76,77,78,79]. Moreover, a study conducted by Vance et al. (2017) [58] discovered the absence of peaks for styrene and acrylonitrile in the Raman aerosol spectra, suggesting that these aerosols were not the result of volatilization but rather likely originated from the VOC photooxidation process. Another example is carbonyl compounds, which can be formed directly through chemical reactions during the printing process or indirectly via ozonolysis of alkene [80,81].

In the above comparisons, the filament composition, printing conditions, and environmental parameters (e.g., sunlight) are the main factors that influence the emissions of VOCs. In order to reduce emissions in a real 3D printing environment, specific mitigation measures may include adjusting the printing temperature (in the range of 200–250 °C), minimizing photo-oxidation in the printing environment, or decreasing gas-phase free radical concentrations through a catalytic filtration system.

## 4. Toxicological Concerns

Previous studies have reported that exposure to 3D printer emissions can induce potential toxic effects in humans or animals, such as respiratory symptoms, inflammation, oxidative stress responses, and cardiovascular impacts [13,14,15,82,83]. Moreover, similar results were found in in vitro studies; rat alveolar macrophages and human bronchial epithelial cells demonstrated a significant reduction in cell viability and a significant increase in intracellular reactive oxygen species induced by PLA and ABS ME printer emissions [84]. Farcas et al. (2022) [85] found that after 24 h of exposure to ABS filaments, normal human-derived bronchial epithelial cells showed a significant increase in proinflammatory markers, including IL-12p70, IL-13, IL-15, IFN-γ, TNF-α, IL-17A, VEGF, MCP-1, and MIP-1α. In contrast, Uribe-Evheverria and Beiras (2022) [86] proved that the PLA filament was harmless to *Paracentrotus lividus* larvae. However, the presence of additives, such as in the plasticizers, in the filaments has toxic effects.

Common hazardous VOC species detected from the ME printing process using ABS and PLA filaments include (but are not limited to) respiratory irritants (toluene and xylenes), asthmagens and strong irritants (styrene), and carcinogens (toluene, benzene, formaldehyde, acetaldehyde, xylene, and isopropyl alcohol). Table S3 summarizes toxicological data of the frequently detected VOC species from FDM 3D printing that uses ABS and PLA materials, including IARC carcinogenicity classifications, inhalation unit risk (IUR) and the reference concentration (RfC) from the Integrated Risk and Information System (IRIS), OELs from Occupational Safety and Health of the German Social Accident Insurance (IFA), and permissible exposure limits (PELs) from OSHA. In the case of 3D printing with ABS, the main volatile compounds generated during printing are styrene and ethylbenzene. Styrene was found to be released from ABS pellets and formed, resulting from the thermal depolymerization of ABS [54]. Acute exposure to styrene can irritate the eyes and respiratory tract and affect the central nervous system, causing depression symptoms such as dizziness and headaches. Long-term exposure may lead to liver damage, peripheral neuropathy, and endocrine problems [54], and styrene has been classified by the IARC as a Group 2A possible human carcinogen [87,88]. The main toxic mechanism involves the metabolic formation of styrene-7,8-oxide, which binds to DNA and induces mutations while simultaneously generating reactive oxygen species (ROS) that trigger oxidative stress and cellular structural damage. Byrley et al. (2020) [22] reported that styrene mass concentrations (0.4 mg/m^3^ and 0.8 mg/m^3^ for 3 min and 5 min samples, respectively) emitted from ABS pellets were close to the EPA IRIS RfC value for inhaled exposure of styrene (1.0 mg/m^3^). Ethylbenzene has been identified in most ABS studies. Acute exposure to ethylbenzene can irritate the eyes and respiratory tract and affect the central nervous system, causing depression symptoms, such as dizziness and headaches [54]. Long-term exposure may lead to abnormalities in liver and kidney function, and it has been classified by the IARC as a Group 2B possible human carcinogen [87,89]. The main toxic mechanism involves liposolubility, which disrupts the integrity of cell membranes, and its metabolites (such as phenylacetaldehyde) consume glutathione, triggering oxidative stress and DNA damage. Other common VOC species found in ME printing that uses ABS include toluene, acetaldehyde, xylene, and benzene (Table S3). All of these substances are considered toxic and harmful to human health. It is also worth considering substances such as acetone or ethanol, which can be detected as process emissions and from the residues after cleaning the print bed. Besides toluene, acetone, and acetaldehyde, other hazardous compounds, including isopropyl alcohol, are commonly detected in PLA printing (Table S3). Isopropyl alcohol is considered an air pollutant due to its high toxicity to the ecological system and carcinogenicity to human health; it is harmful to the central nervous system, eyes, nose, throat, and lungs [88]. The acute effects of exposure to high concentrations of acetonitrile include irritation of mucous membranes; however, the concentrations detected in the ME printing process using PLA were much lower than the recommended safe exposure limits (70 mg/m^3^ in OSHA TWA) [90]. This finding indicates that laboratory workers were not at risk of acute toxicity effects, but further studies should be conducted on the effects of prolonged exposure to acetonitrile concentrations.

Furthermore, various VOC species can be directly emitted or form secondary pollutants through the printing process, thereby increasing the impact on human health. The level of VOC emissions can be significantly enhanced, leading to higher human exposure risks [12]. Based on VOC substances measured in the indoor printing environment (e.g., the laboratory and printing room), the worst-case values of 15 hazardous compounds were selected for potential health risk assessment of targeted individuals (Table 2). The chronic daily intake and lifetime hazardous cancer risk are determined by the frequency, duration, and activity patterns of inhalation exposure. The equations and details of inhalation exposure parameters are listed in the Supplementary Materials (Table S4). As shown in Table 2, the cancer risk for 3D printing workers ranged from 1.79 × 10^−6^ (acetaldehyde) to 2.11 × 10^−4^ (ethylbenzene). Although ethylbenzene is less commonly detected in 3D printing environments, benzene, ethylbenzene, acetaldehyde, and formaldehyde are carcinogenic to humans when inhaled. The total cancer risk was estimated to be 2.40 × 10^−4^ (larger than 10^−6^), which is considered a potential carcinogenic concern. For VOC substances that are not defined as carcinogenic compounds but have a health impact, the hazard quotient (HQ) is introduced, and its calculation equation is provided in Table S4, Equation (S2). If the calculated HQ is <1, there is no adverse health effect. According to Table 2, the HQ values for individual VOC species are below 1; when we sum up the individual HQ values, it amounts to 1.2567, indicating that the health impact of long-term exposure to non-carcinogenic VOC substances released during the ME printing processes using ABS and PLA in various printing environment cannot be ignored. Furthermore, regarding the 15 hazardous compounds, most of them are regulated under occupational exposure standards. However, in terms of indoor air quality management, while acetaldehyde has specific regulatory limits in indoor air management standards in China and Japan, six substances—benzene, formaldehyde, toluene, o/p-xylene, ethylbenzene, and styrene—have individual regulatory limits in management documents concerning indoor air quality (VOC emissions and exposure) in Europe, the United States, and China. Acetone, butanone, acetic acid, and isopropyl alcohol may be included in the overall control of TVOCs (total volatile organic compounds). Notably, methyl methacrylate, phenol, and benzoic acid have not yet been included in indoor air quality management limits.

Overall, the evidence mentioned above indicates a possible release of substantial quantities of toxic and carcinogenic VOCs during 3D printing processes. Some of these VOCs were found to be close to the recommended indoor levels associated with adverse health effects [91]. This review emphasizes the necessity for further investigation into the toxicity of air pollutants in 3D printing environments, particularly regarding exposure to mixtures of VOCs and their long-term health implications. Additionally, considering the extensive utilization of ME printing technology in offices and households, it is necessary to consider indoor safety and health principles in 3D printing workplaces [92]. Manufacturers of ME printers should encourage the adoption of best practices for 3D printing operations to minimize emissions during the printing process. These practices may include choosing materials with lower VOC emissions, ensuring proper ventilation and installing local exhaust ventilation systems when working with 3D printers to reduce exposure to harmful VOCs. Additionally, it is important to ensure that workers wear suitable protective equipment to minimize human exposure.

## 5. Summary and Future Perspectives

In the literature, it has been found that numerous VOCs are emitted from ABS and PLA filaments during printing, including styrene, isopropyl alcohol, and ethanol, among others. The raw materials of filaments, additives (i.e., the dye and stabilizer), printing operation temperature, and conditions of the indoor environment affect the composition and concentration of emitted VOCs. We concluded that there are significant variations in VOC emission levels across studies, even when using the same printing materials of the same color. These variations are influenced by (1) analytical methods; (2) testing environments such as a chamber or real indoor environment; (3) the volume of test chambers or workplaces; and (4) the duration of printing operations. Underwriters of Laboratory Chemical Safety and the Georgia Institute of Technology published the “Standard method for testing and assessing particle and chemical emissions from 3D printers” [93]. This standardized method and standard protocols enable the collection of comparable measurement data in various research results. However, studies on the characteristics of VOC emissions during 3D printing using this standardized method are still limited. In addition, due to the limited analytical techniques, the number of VOC categories emitted from 3D printing remains largely unknown. Based on real indoor measurement studies, it is evident that most of the emitted VOCs can be inhaled, and their concentrations could cause health risks for workers or users exposed over the long term. The identified VOCs, i.e., benzene, formaldehyde, and acetaldehyde, are considered carcinogenic compounds, indicating a potential carcinogenic concern with a total cancer risk of 2.40 × 10^−4^. Exposure to styrene, isopropyl alcohol, xylene, and other substances leads to potential non-carcinogenic health impacts for workers. Therefore, several suggestions and recommendations are presented: (1) apart from standardizing measurement methods and protocols, appropriate strategies and policies for controlling VOCs from printers and filament manufacturing are necessary. (2) Proper guidance for improving and ensuring good indoor ventilation is needed to reduce exposure levels. (3) Best practices of 3D printer operation are needed to minimize harmful pollutants. (4) It is necessary to incorporate additional pollutant parameters into the occupational safety regulatory framework.

Furthermore, there are still many unanswered questions regarding the characteristics of VOC emissions from 3D printers. These include (1) identifying the types of VOCs emitted by different types of 3D printers and printing filaments available in the current market using high-throughput screening methods; (2) in addition to temperature, determining how other environmental factors, such as humidity, affect VOC emissions from 3D printing; (3) investigating secondary formation processes or emitted VOCs that interact with common indoor gaseous or particulate pollutants like O_3_, OH, nitrate, sulfate, etc.; and (4) assessing the overall impact of mixtures of VOCs released from 3D printers on human health and indoor environments. Therefore, additional experimental studies are needed in the future to obtain a comprehensive understanding of the VOC emission characteristics of 3D printers.

## Figures and Tables

**Table 1:** VOC species emissions from red ABS filaments.

μg/m^3^	Printer 1 ^a^	Printer 2 ^b^	Printer 3 ^c^
styrene	912.8	237.1	17.0
ethylbenzene	59.9	6.6	13.0
acetophenone	61.5		2.0
benzene		0.4	
acetaldehyde		7.7	
isopropyl alcohol		108.1	
ethanol		39.9	
acetone		31.5	
xylene		3.0	
benzaldehyde			6.0
c14(tetradecane)			2.0
acetic acid			3.0
phenol			1.0
hexadecane			2.0
toluene	40.4		
hexanal	70.5		
octanal	41.4		
nonanal	39.7		
1-butano	39.5		
benzenemethanol, alpha., alpha.-di	44.8		
alpha.-pinene	42.2		

Remark: ^a^ LulzBot Mini (U.S.A) (extruder temp: 240 °C, bed temp: 110 °C); ^b^ MakerBot 2x (extruder temp: 230 °C, bed temp: 110 °C); ^c^ Zortrax (Poland) (extruder temp: 240 °C, bed temp: 80 °C); [41,43,44].

**Table 2:** Calculation result of cancer and non-cancer risk of exposure to selected VOCs.

Compounds	Classification	Exposure Concentration (EC, μg/m^3^)	Cancer Risk (CR)	Hazardous Quotient (HQ)
**benzene**	1	0.51	3.97 × 10^−6^	0.0170
**ethylbenzene**	2B	84.26	2.11 × 10^−4^	0.0842
**formaldehyde**	1	1.83	2.38 × 10^−5^	0.0062
**acetaldehyde**	2B	0.81	1.79 × 10^−6^	0.0905
**styrene**	2A	26.46		0.0265
**isopropyl alcohol**	3	142.47		0.7123
**methyl methacrylate**	3	1.93		0.0028
**phenol**	3	0.92		0.0000
**toluene**	3	0.51		0.0001
**o-xylene**	/	29.51		0.2951
**p-xylene**	/	0.61		0.0204
**acetic acid**	/	1.32		0.0001
**butanone**	/	0.81		0.0002
**acetone**	/	11.19		0.0004
**benzoic acid**	/	1.93		0.0010
**Total cancer risk**			**2.40 × 10^−4^**	
**Total hazard quotient (HQs)**				**1.2567**

## Data Availability

This is a review paper where no new data were created.

## Supplementary Materials

The following supporting information can be downloaded at: https://www.mdpi.com/article/10.3390/toxics13040276/s1. Table S1. VOC emission rates (μg/min) during ME printing using different ABS filaments; Table S2. VOC emission rates (μg/min) during ME printing using different PLA filaments; Table S3. Toxicological data and worst-case VOC concentration measured in real indoor environment; Table S4. Exposure parameters of adult workers and equations of exposure concentration (EC), non-cancer risk (HQ), and cancer risk (CR). [13,21,35,38,39,41,42,44,45,47,48,49,50,51,50,51,52,53,54,55,56,57,58,59,60,61,87,89].
