# Supplementary material for: Emission Characteristics of Volatile Organic Compounds from Material Extrusion Printers Using Acrylonitrile–Butadiene–Styrene and Polylactic Acid Filaments in Printing Environments and Their Toxicological Concerns
Source: Toxics. 2025 Apr 4;13(4):276. doi: 10.3390/toxics13040276 (PMC12031625; doi:10.3390/toxics13040276)
Supplement: Supplementary file 1 [file toxics-13-00276-s001.zip › toxics-3544840-supplementary.pdf]

Supplementary Materials

Emission Characteristics of Volatile Organic Compounds from Material Extrusion Printers Using Acrylonitrile–Butadiene–Styrene and Polylactic Acid Filaments in Printing Environments and Their Toxicological Concerns

Yuan Gao [1], Yawei Xue [1,2], Chenyang Sun [2,3,4,5], Luhang She [2,3,4,5] and Ying Peng [2,3,4,5,*]

[1] Instrumentation and Service Center for Science and Technology, Beijing Normal University, Zhuhai 519087, China; yuan.gao@bnu.edu.cn (Y.G.); 202321180070@mail.bnu.edu.cn (Y.X.)
[2] Research and Development Center for Watershed Environmental Eco-Engineering, Advanced Institute of Natural Sciences, Beijing Normal University, Zhuhai 519087, China; 15893863095@163.com (C.S.); 15764410923@163.com (L.S.)
[3] State Key Laboratory of Wetland Conservation and Restoration, School of Environment, Beijing Normal University, Beijing 100875, China
[4] Key Laboratory of Coastal Water Environmental Management and Water Ecological Restoration of Guangdong Higher Education Institutes, Beijing Normal University, Zhuhai 519087, China
[5] Zhuhai Key Laboratory of Coastal Environmental Processes and Ecological Restoration, Beijing Normal University, Zhuhai 519087, China
* Correspondence: pengying@bnu.edu.cn

Table of contents

Table S1. VOC emission rates (μg/min) during ME printing using different ABS filaments.

Table S2. VOC emission rates (μg/min) during ME printing using different PLA filaments.

Table S3. Toxicological data and worst-case VOCs concentration measured in real indoor environment.

Table S4. Exposure parameters of adult worker and equations of exposure concentration (EC), non-cancer risk (HQ) and cancer risk (CR)

Table S1. VOC emission rates (µg/min) during ME printing using different ABS filaments

**µg/min**	**red**[a]	**red**[b]	**red**[c,k]	**red**[d]	**red**[e]	**blue**[a,k]	**blue**[b]	**white**[b,i]	**white**[b,j,k]	**gray**[a]	**green**[a]	**black**[c]	**natural**[c]	**ABS**[f]	**ABS**[g]	**ABS**[e]	**ABS**[h]
styrene	6.41	54.21	8.47	12.11	4.81		11.51	33.5	113.10			3.59	9.00	0.29	4.60	20.50	
ethylbenzene	4.81	3.41	0.24		2.51			2.11	5.42				0.17	0.23			
acetone			1.13							0.41		1.62	0.54	0.01			
acetaldehyde			0.28								0.41	0.40	0.58		0.89		
acetophenone		3.71			2.51		1.11		7.51						1.05	9.50	
Ethanol			1.43							0.21		2.25	2.05				
1-butanol		2.41							4.00						0.33		
xylene			0.11										0.01		0.11		
decanal							0.11		5.81						0.11		
octanal		0.11					0.3	0.41									
Isopropyl alcohol			3.86									7.61	3.11				
benzaldehyde	0.81														1.19		
benzene			0.01									0.04					
toluene		0.81													0.10		
2-propenal						0.3				0.31							
hexanal							0.81	1.91									
nonanal		0.11						1.11									
1R,alpha,-pinene		1.11					0.61										
butadien														0.08			
cyclohexanone														0.11			
iso-butanol														0.06			
formaldehyde				2.41											0.41		0.13
cumene															0.30		

vinyl cyclohexene						0.34		2.69
acrylonitrile					0.21			4.65
bicyclo(3,1,1)hept-2-ene, 2,6,6-tri			0.21					
propylene glyco		7.31						
glycerin		1.81						
1-pentanol		1.51						
benzenemethanol, alpha., alpha.-di	2.71							
isopropyl palmitate				6.42				
tetrachloroethylene				5.51				
dl-2-phenyl-1,2-propanediol				5.51				
cyclotrisiloxane, hexamethyl				5.32				
benzenethanamine,N-[(phentafi				4.11				
alpha-methylstyrene							21.50	

Sources: [a] Gu et al. 2019 [42]; [b] Azimi et al. 2016 [44]; [c] Stefaniak et al. 2017 [41]; [d] Mendes et al., 2017 [36]; [e]Floyd et al., 2017 [47].; [f] Wojtyła et al., 2020 [51]; [g] Davis et al., 2019 [21]; [h] Zisook et al. 2020 [45]; [k]Zhang and Black, 2023 [49]

[i] FlashForge printer with while colour filament

[j] MarkerBot printer with white colour filament

Table S2. VOC emission rates (μg/min) during ME printing using different PLA filaments

**μg/min**	**red**[a]	**red**[b,h]	**red**[c]	**green**[a]	**ocean blue**[a]	**trans blue**[a]	**white**[b,h]	**yellow**[c,h]	**bronze**[c]	**orange**[d]	**PLA**[e]	**PLA**[f]	**PLA**[g]
acetone	0.96			1.14	0.26	0.13							
acetaldehyde	0.13			0.20	0.18	0.18					0.01		0.14
toluene			0.82	0.07	0.04		1.13					0.31	
acetonitrile	0.19			0.01	0.01	0.04							
ethanol	3.69			3.04	1.98	2.61							
isopropyl alcohol	19.73			56.53	9.94	3.55							
nonanal		0.51	0.67				0.59						0.36
octanal		0.49	0.79				0.71					0.05	
hexanal		0.64	1.57				1.63						
1R-alpha,pinene		0.35	0.51				0.54						
1,4-dioxane-2,5-dione, 3,6-dimethyl		5.00	4.40				3.70	6.00	11.00				
acrylic acid													
methyl-methacrylate													
isopropyl palmitate		1.29	0.54								0.25	0.55	
ethanol, 2-(2-butoxyethoxy)-			2.30				1.60						
styrene			0.62										
tetrachloroethylene		0.45										0.03	
2-heptanone, 3-methyl-							0.56						
cyclohexanone													

1-butanol					0.10		
isobutanol						0.30	
formaldehyde				2.40	0.11		
benzaldehyde						0.12	
caprolactam						0.07	
cyclotrisiloxane						0.12	
1-hexanol, 2-ethyl						0.01	
benzene, ethyl						0.01	
2,4-bis(1,1-dimethylethyl)-phenol						0.01	
12-crown-4	2.00					0.14	
chloromethyl methl sulfide			2.50				
1-propanol, 2-ethoxy			2.10				
benzene, 1,3-dimethyl	0.40						
2-butanone		0.57					
propylene glycol	0.30						
lactide							1.07
decanal						1.85	0.43
acetic acid						0.07	0.25
benzoic acid							0.18

Sources: [a] Stefaniak et al. 2017 [41]; [b] Azimi et al. 2016 [44]; [c] Floyd et al., 2017 [47]; [d]Mendes et al., 2017 [36]; [e] Wojtyła et al. 2017 [50]; [f] Davis et al. 2019 [21]; [g] Hall et al. 2019 [48], [h]Zhang and Black, 2023 [49]

Table S3. Toxicological data and worst-case VOCs concentration measured in real indoor printing environment.

VOCs species	CAS No.	IARC [a] human carcinogenicity classifications	IRIS [b] Inhalation unit risk (IUR, m^3 /μg)	IRIS [b] Reference concentration for Inhalation Exposure (RfC, mg/m^3)	Germany IFA[c] Occupational exposure limits（8 hours）（OELs, mg/m^3)	OSHA [d] (limits for Air Contaminants) PEL [e] (8-hour TWAs [f]) (mg/m^3)	Worst case concentration ($\mu g/m^3$) [g]
Benzene	71-43-2	1	2.20E-06	0.03	1.9 [h]	3.19	5
Formaldehyde	50-00-0	1	1.30E-05	0.007	0.37	0.92	18
Acetaldehyde	75-07-0	2	2.20E-06	0.009	91	180	8
Ethylbenzene	100-41-4	2B	/	1	87	435	828
Styrene	100-42-5	2A	/	1	86	215	260
Isopropyl alcohol	67-63-0	3	/	0.2	500 [h]	980	1400
Methyl methacrylate	80-62-6	3	/	0.7	210	/	19
Phenol	108-95-2	3	/	96.2	8 [h]	/	9
Toluene	108-88-3	3	/	5	190 [h]	375	5
o-xylene	1330-20-7	/	/	0.1	220 [h]	435	290
p-xylene	106-42-3	/	/	0.03	220 [h]	435	6
Acetic acid	64-19-7	/	/	10	25	/	13
2-Butanone	78-93-3	/	/	5	600 [h]	590	8
Acetone	67-64-1	/	/	30.881	1200	1800	110
benzoic acid	65-85-0	/	/	1.9	0.5 [h]	/	19

Sources: (IARC, 2000; IARC, 2019 [87,89]) https://www.osha.gov/); IRIS (https://www.epa.gov/iris); NIOSH (https://www.cdc.gov/niosh); Chan et al., 2020 [13]; Steinle 2016 [38]; Väisänen, et al. 2019 [38]; Dobrzynska et al., 2022 [61]; Khaki et al., 2022 [60]; Chuang et al., 2024 [59]

[a] IARC: International Agency for Research on Cancer (https://www.iarc.who.int)

[b] IRIS: Integrated Risk and Information System (https://www.epa.gov/iris)

[c] Germany IFA: Occupational Safety and Health of the German Social Accident Insurance (https://limitvalue.ifa.dguv.de/WebForm_ueliste2.aspx)

[d] OSHA: Occupational Safety and Health Administration (https://www.cdc.gov/niosh/npg/nengapdxg.html)

[e] PEL: Permissible exposure limits

[f] TWA: The maximum time-weighted average

[g] data summarized from the studies of Chan et al. 2020 [13]; Steinle 2016 [38]; Väisänen, et al. 2019 [39]

[h] 15 minutes average value

Table S4. Exposure parameters of adult worker and equations of exposure concentration (EC), non-cancer risk (HQ) and cancer risk (CR)

	**Exposure time (ET)**	**Exposure frequency (EF)**	**Exposure duration (ED)**	**Average time (AT)**
	**(hours/day)**	**(days/year)**	**(years/life)**	**(hours/life)**
**Adult (exposure parameters)**	8	260<br>(assume 5-day work/week))	30<br>(assume 30 years work)	613200<br>(assume 70 years life)
**Exposure concentration (EC)**[a]	EC = $\frac{VOCs\ concentration\ ((\mu g/m^3)\times ET\left(\frac{h}{day}\right)\times EF(\frac{day}{year})\times ED(\frac{year}{life})}{AT\ (\frac{h}{life})}$ Eq. (S1)			
**Non-cancer risk calculation (HQ)**[b]	HQ = $\frac{EC\left(\frac{mg}{m^3}\right)}{RfC\ (\frac{mg}{m^3})}$ Eq. (S2)			
**Cancer risk calculation (CR)**[c]	CR = EC ($\mu g/m^3$) × IUR $(\mu g/m^3)^{-1}$ Eq. (S3)			

[a-c] the equation is referred to Part F, Supplemental Guidance for inhalation risk assessment of human health evaluation manual of risk assessment guidance U.S. EPA (https://semspub.epa.gov/work/HQ/140530.pdf.)

References:

13. Chan FL, House R, Kudla I, Lipszyc JC, Rajaram N, Tarlo SM. (2018) Health survey of employees regularly using 3D printers. Occup Med (Lond); 68: 211-214.

21. Davis AY, Zhang Q, Wong JPS, Weber RJ, Black MS. (2019) Characterization of volatile organic compound emissions from consumer level material extrusion 3D printers. Build Environ. 160: 106209

35. Finnegan, M., C. L. Thach, S. Khaki, E. Markey, D. J. O'Connor, A. F. Smeaton, A. Morrin. (2023). Characterization of volatile and particulate emissions from desktop 3d printers. Sensors 23:9660.

36. Mendes L, Kangas A, Kukko K, Mølgaard B, Säämänen A, Kanerva T, et al. (2017) Characterization of Emissions from a Desktop 3D Printer. Journal of Industrial Ecology; 21: S94-S106.

38. Steinle P. (2016) Characterization of emissions from a desktop 3D printer and indoor air measurements in office settings. Journal of Occupational and Environmental Hygiene; 13: 121-132

39. Väisänen AJK, Hyttinen M, Ylönen S, Alonen L. (2019) Occupational exposure to gaseous and particulate contaminants originating from additive manufacturing of liquid, powdered, and filament plastic materials and related post-processes. J Occup Environ Hyg; 16: 258-271.

41. Stefaniak AB, LeBouf RF, Yi J, Ham J, Nurkewicz T, Schwegler-Berry DE, et al. (2017) Characterization of chemical contaminants generated by a desktop fused deposition modeling 3-dimensional Printer. J Occup Environ Hyg. 14: 540-550.

42. Gu J, Uhde E, Wensing M, Xia F, Salthammer T (2019a) Emission Control of Desktop 3D Printing: The Effects of a Filter Cover and an Air Purifier. Environ. Sci. Technol. Lett. 6: 499-503.

44. Azimi P, Zhao D, Pouzet C, Crain NE, Stephens B. (2016) Emissions of Ultrafine Particles and Volatile Organic Compounds from Commercially Available Desktop Three-Dimensional Printers with Multiple Filaments. Environ. Sci. Technol. 50: 1260-1268.

45. Zisook RE, Simmons BD, Vater M, Perez A, Donovan EP, Paustenbach DJ, et al. (2020)Emissions associated with operations of four different additive manufacturing or 3D printing technologies. J Occup Environ Hyg. 17: 464-479.

47. Floyd EL, Wang J, Regens JL. (2017) Fume emissions from a low-cost 3-D printer with various filaments. Journal of occupational and environmental hygiene; 14: 523-533.

48. Hall S, Penegelly I, Staff J (2019) Measuring and controlling emissions from polymer filament desktop 3D printers. Research Reports, RR1146.

49. Zhang Q, Black MS. (2023) Exposure hazards of particles and volatile organic compounds emitted from material extrusion 3D printing: Consolidation of chamber study data. Environment International; 182.

50. Wojtyła S, Klama P, Baran T. (2017) Is 3D printing safe? Analysis of the thermal treatment of thermoplastics: ABS, PLA, PET, and nylon. J Occup Environ Hyg; 14: 80-

85.
51. Wojtyła S, Klama P, Śpiewak K, Baran T. (2020) 3D printer as a potential source of indoor air pollution. Int J Environ Sci Technol; 17: 207-218.
59. Chuang Y-S, Berekute AK, Hsu H-Y, Wei H-S, Gong W-C, Hsu Y-Y, et al. 2024. Assessment of emissions and exposure in 3D printing workplaces in Taiwan. Journal of Occupational and Environmental Hygiene; 21: 270-286.
60. Khaki S, Rio M, Marin P. Characterization of Emissions in Fab Labs: An Additive Manufacturing Environment Issue. Sustainability 2022; 14.
61. Dobrzynska E, Kondej D, Kowalska J, Szewczynska M. (2022) Exposure to chemical substances and particles emitted during additive manufacturing. Environmental Science and Pollution Research; 29: 40273-40278.
87. IARC. (2000) Evaluation of Carcinogenic Risks to Humans: some industrial chemicals. 15-22 February 2000, Lyon, France. IARC Monogr Eval Carcinog Risks Hum; 77: 1-529.
89. IARC. IARC Monographs on the Evaluation of Carcinogenic Risks to Humans: Styrene, Styrene-7,8-oxide, and Quinoline. Styrene, Styrene-7,8-oxide, and Quinoline. International Agency for Research on Cancer
